# Supplementary material for: Lipid-polymer nanoparticles to probe the native-like environment of intramembrane rhomboid protease GlpG and its activity
Source: Nat Commun. 2024 Aug 30;15:7533. doi: 10.1038/s41467-024-51989-0 (PMC11364529; doi:10.1038/s41467-024-51989-0)
Supplement: Supplementary file 1 — Supplementary Information [file 41467_2024_51989_MOESM1_ESM.pdf]

Supplementary Information

Lipid-polymer nanoparticles to probe the native-like environment of intra-membrane rhomboid protease GlpG and its activity

Henry Sawczyc[1], *Takashi Tatsuta*[2], *Carl Öster*[1], *Spyridon Kosteletos*[1], *Sascha Lange*[1], *Claudia Bohg*[1], *Thomas Langer*[2], *Adam Lange*[1,3,*]

[1] Research Unit Molecular Biophysics, Leibniz-Forschungsinstitut für Molekulare Pharmakologie, Robert-Rössle-Straße 10, 13125 Berlin, Germany

[2] Max-Planck-Institute for Biology of Ageing, Department of Mitochondrial Proteostasis, Joseph-Stelzmann-Str. 9b, 50931 Cologne, Germany

[3] Institut für Biologie, Humboldt-Universität zu Berlin, Invalidenstraße 42, 10115 Berlin, Germany

* to whom correspondence should be addressed (alange@fmp-berlin.de)

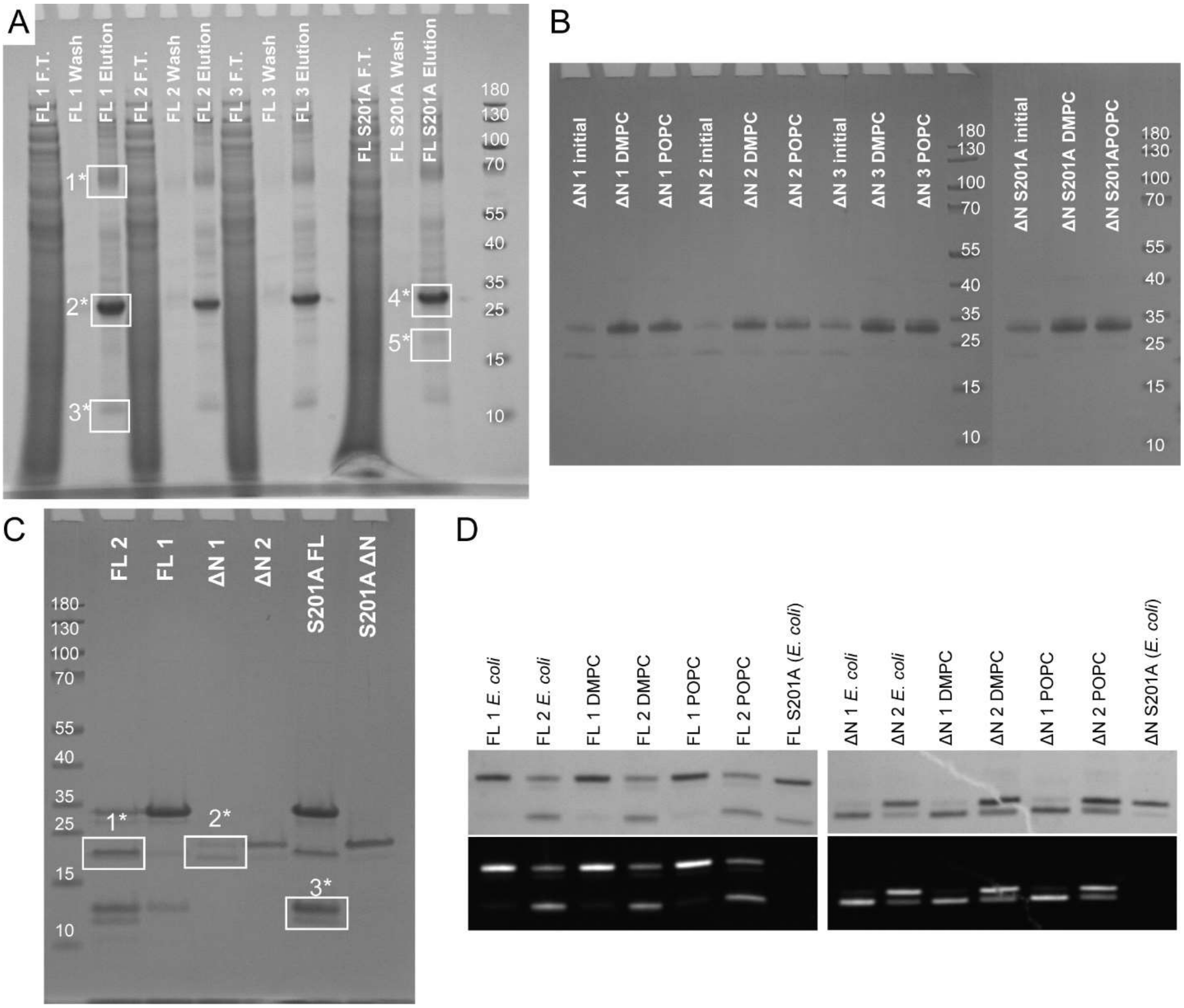

Supplementary Figure 1 – Nu-PAGE (SDS-PAGE) gels of: (A) Ni-NTA purification of GlpG solubilized by DIBMA, showing unbound (F.T.), wash, and elution fractions per sample and S201A inactive mutant, numbered samples indicate biological replicates. Numbered boxes indicate samples sent for trypsin

digest identification, where the primary proteins identified are: 1 – 60kDa chaperonin (groL), 2 – **GlpG**, 3 – Uncharacterized protein YjeL, 4 – GlpG (S201A), 5 – Peptidogycan-associated lipoprotein (pal). (B) GlpGΔN purity assessment, where numbered samples indicate biological replicates. (C) Purity of eluted fractions of DM-solubilized GlpG (FL 1, 2) and GlpGΔN (ΔN 1,2), where 1 and 2 represent biological replicates, and associated catalytically inactive S201A mutants. Numbered boxes indicate samples sent for trypsin digest identification, where the primary proteins identified are: 1 – **GlpGΔN**, 2 – 50S ribosomal protein L13 (rplM), 3 – 30S ribosomal protein S15 (rpsO). (D) TAMRA-FP labelling of DM-solubilized GlpG (FL), GlpGΔN (ΔN), and S201A inactive mutants, reconstituted into different lipid environments (*E. coli* total lipid extract, DMPC, POPC). Numbering indicates biological repeats

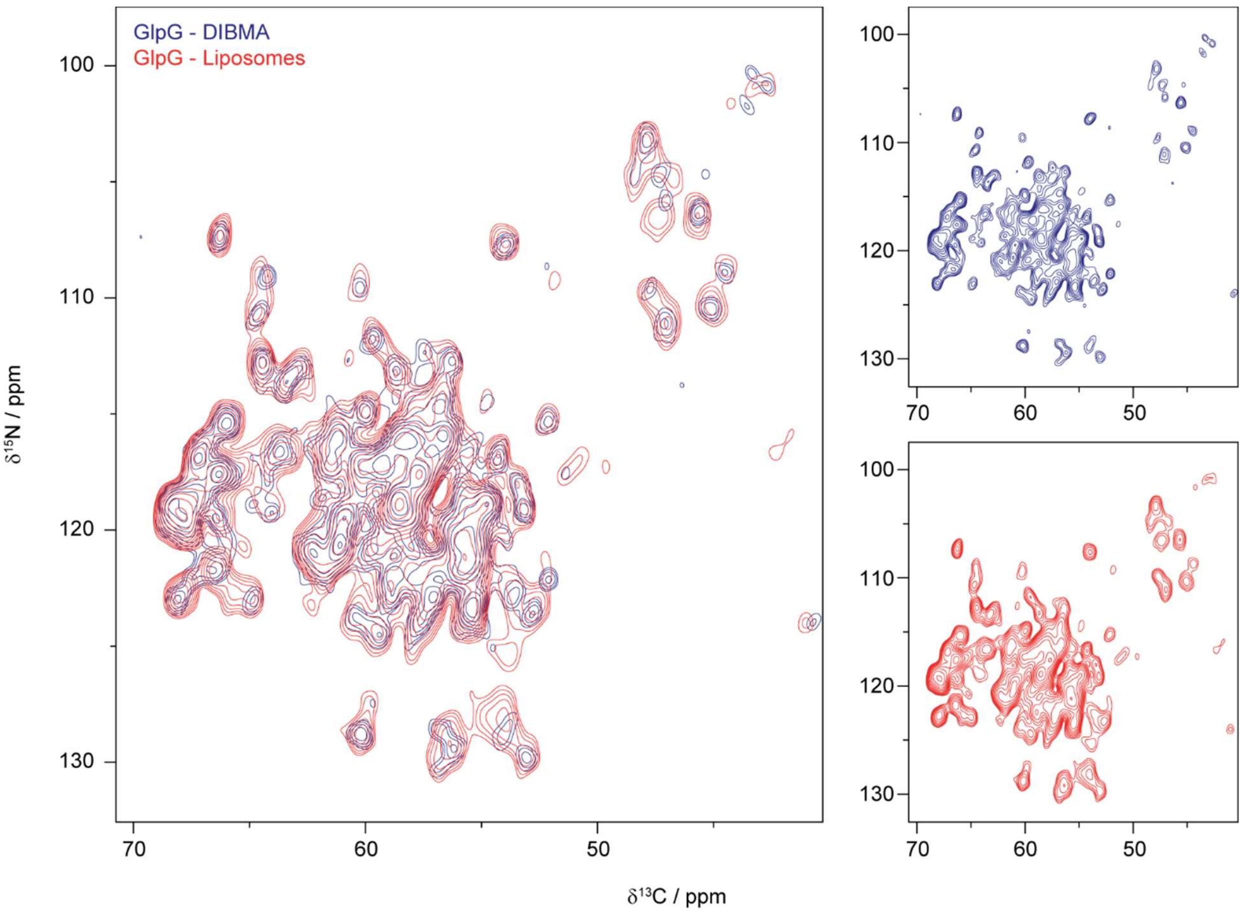

Supplementary Figure 2 – ^{13}C detected 2D hNCα spectra of ^{13}C,^{15}N-labelled GlpG in native DIBMALPs (blue) and in *E.coli* total lipid extract liposomes (red). The spectrum of GlpG DIBMALPs was recorded on a 700 MHz spectrometer with 35 kHz magic-angle spinning (1.9 mm probe) and at a sample temperature of 20˚C. The spectrum of GlpG in liposomes was recorded on a 600 MHz spectrometer with 40 kHz magic-angle spinning (1.9 mm probe) and at a sample temperature of 23˚C.

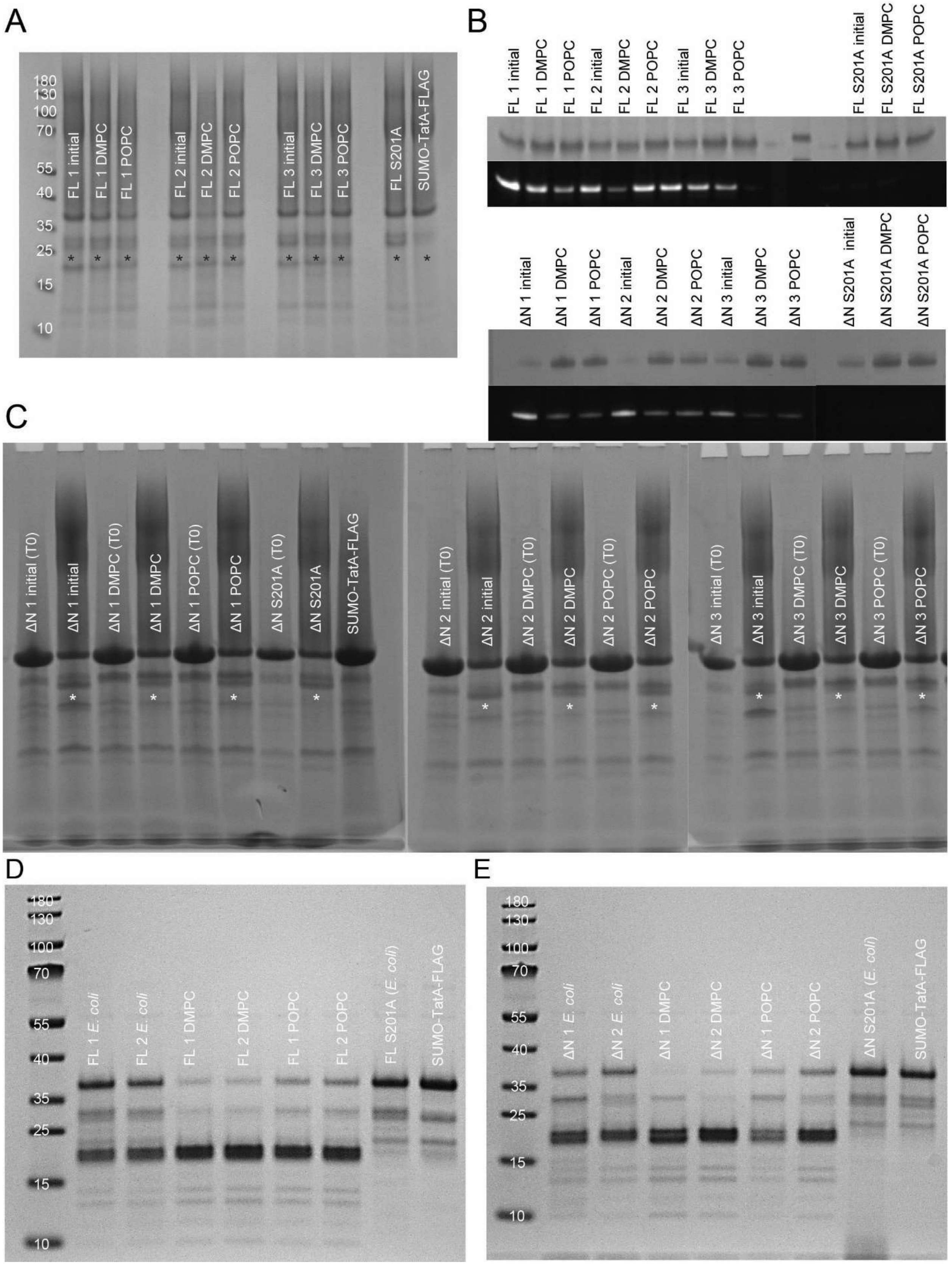
A
180
130
100
70
55
40
35
25
15
10
FL 1 initial
FL 1 DMPC
FL 1 POPC
FL 2 initial
FL 2 DMPC
FL 2 POPC
FL 3 initial
FL 3 DMPC
FL 3 POPC
FL S201A
SUMO-TatA-FLAG
B
FL 1 initial
FL 1 DMPC
FL 1 POPC
FL 2 initial
FL 2 DMPC
FL 2 POPC
FL 3 initial
FL 3 DMPC
FL 3 POPC
FL S201A initial
FL S201A DMPC
FL S201A POPC
ΔN 1 initial
ΔN 1 DMPC
ΔN 1 POPC
ΔN 2 initial
ΔN 2 DMPC
ΔN 2 POPC
ΔN 3 initial
ΔN 3 DMPC
ΔN 3 POPC
ΔN S201A initial
ΔN S201A DMPC
ΔN S201A POPC
C
ΔN 1 initial (T0)
ΔN 1 initial
ΔN 1 DMPC (T0)
ΔN 1 DMPC
ΔN 1 POPC (T0)
ΔN 1 POPC
ΔN S201A (T0)
ΔN S201A
SUMO-TatA-FLAG
ΔN 2 initial (T0)
ΔN 2 initial
ΔN 2 DMPC (T0)
ΔN 2 DMPC
ΔN 2 POPC (T0)
ΔN 2 POPC
ΔN 3 initial (T0)
ΔN 3 initial
ΔN 3 DMPC (T0)
ΔN 3 DMPC
ΔN 3 POPC (T0)
ΔN 3 POPC
D
180
130
100
70
55
40
35
25
15
10
FL 1 E. coli
FL 2 E. coli
FL 1 DMPC
FL 2 DMPC
FL 1 POPC
FL 2 POPC
FL S201A (E. coli)
SUMO-TatA-FLAG
E
180
130
100
70
55
40
35
25
15
10
ΔN 1 E. coli
ΔN 2 E. coli
ΔN 1 DMPC
ΔN 2 DMPC
ΔN 1 POPC
ΔN 2 POPC
ΔN S201A (E. coli)
SUMO-TatA-FLAG

Supplementary Figure 3 – Nu-PAGE (SDS-PAGE) gels showing GlpG and GlpG∆N activity. (A) Cleavage of DIBMA-solubilized SUMO-TatA-FLAG (~35 kDa) to ~17 kDa components by wild type or full-length (FL) GlpG in natively extracted membrane ('initial'), post DMPC wash ('DMPC'), or post POPC wash ('POPC'). A * marking highlights expected mass of cleaved product. Numbers indicate biological repeats. (B) TAMRA-FP labelling of wild type (FL) GlpG, GlpG∆N, and associated inactive S201A mutants solubilized by DIBMA into natively extracted membrane DIBMALPs ('initial'), post DMPC wash ('DMPC'), or post POPC wash ('POPC'). Numbering indicates biological repeats (C) Cleavage of DIBMA-solubilized SUMO-TatA-FLAG (~35 kDa) to ~17 kDa components by GlpG∆N in natively extracted membrane ('initial'), post DMPC wash ('DMPC'), or post POPC wash ('POPC'). T0 indicates initial starting composition for named sample, and * highlights expected mass of cleaved product. Sample numbering indicates biological repeats. (D) Cleavage of SUMO-TatA-FLAG (~35 kDa) to ~17 kDa components by wild type or full-length (FL) GlpG solubilized by DM and reconstituted into *E. coli* total lipid extract (*E. coli*), DMPC, or POPC liposomes. Numbers indicate biological repeats. (E) Cleavage of SUMO-TatA-FLAG (~35 kDa) to ~17 kDa components by GlpG∆N solubilized by DM and reconstituted into *E. coli* total lipid extract (*E. coli*), DMPC, or POPC liposomes. Numbers indicate biological repeats.

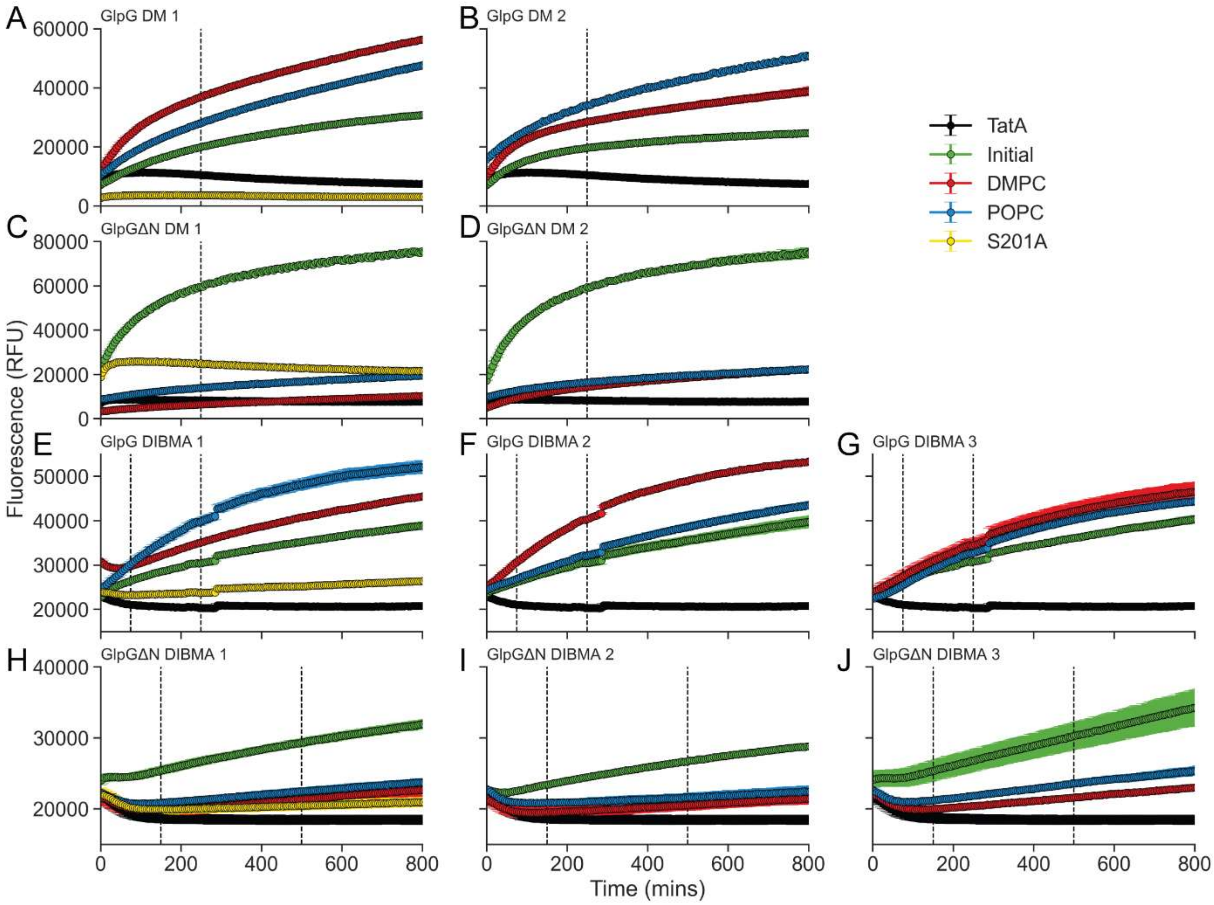

Supplementary Figure 4 – Raw fluorescence measurement data for TatA-FITC cleavage by DM-solubilized GlpG (A, B) and GlpGΔN (C, D) reconstituted into liposomes and GlpG (E-G), and GlpGΔN (H-J) solubilized into DIBMALPs. Colors (top right) denote sample identity, where respective S201A mutant is only shown in left-hand side samples. For DM-solubilized and reconstituted samples, green denotes not initial but *E. coli* total lipid extract. Errors shown are standard deviation of the mean, from technical repeats (n=3). Vertical dotted lines denote region chosen for linear regression. For the DM controls (A-D), only the rates calculated from A and C are represented in Figure 4.

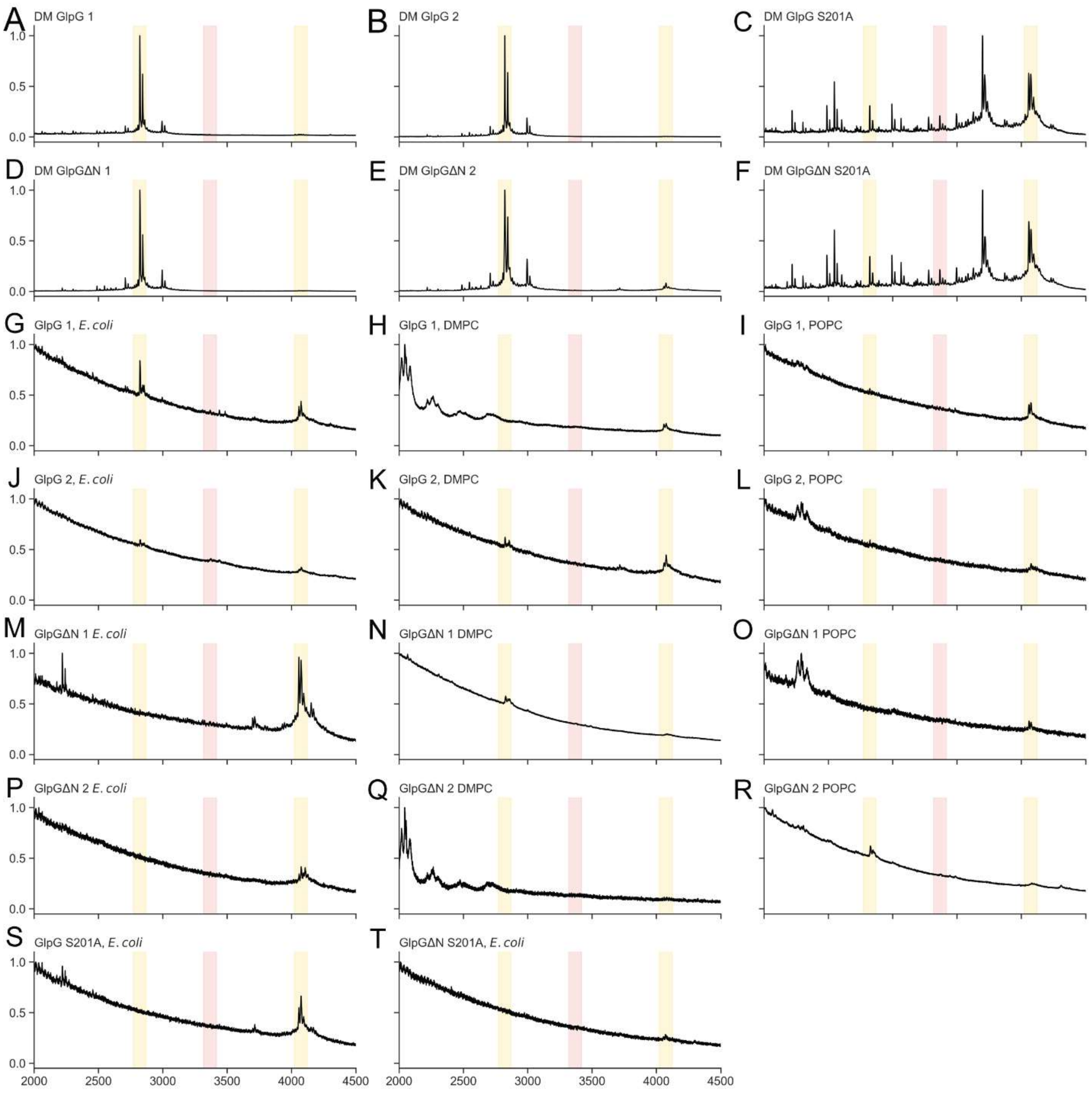

Supplementary Figure 5 – Normalized MALDI-TOF spectra after 24 hours incubation of TatA:GlpG in a 5:1 molar ratio from DM-solubilized GlpG and GlpGΔN. (A-F) MALDI spectra of TatA-FITC cleavage by GlpG (A-C) and GlpGΔN (D-F) in DM detergent, with (C) and (F) corresponding to the respective inactive S201A mutant. (G-T) MALDI spectra of TatA-FITC cleavage by GlpG (G-L, S) and GlpGΔN (M-R,T) reconstituted into liposomes (*E. coli* total lipid extract, DMPC, or POPC). Numbering indicates biological repeats. Yellow highlighted regions denote expected regions of either cleaved TatA peptide (left, ~2800 Da), or un-cleaved TatA-FITC (right, ~4100 Da). Red highlighted region denotes unexpected cleavage product, with a calculated mass of 3368 Da, shown more clearly in DIBMA samples (Supplementary Fig. 6). S and T show MALDI spectra for TatA incubated with inactive mutant S201A of GlpG and GlpGΔN, respectively.

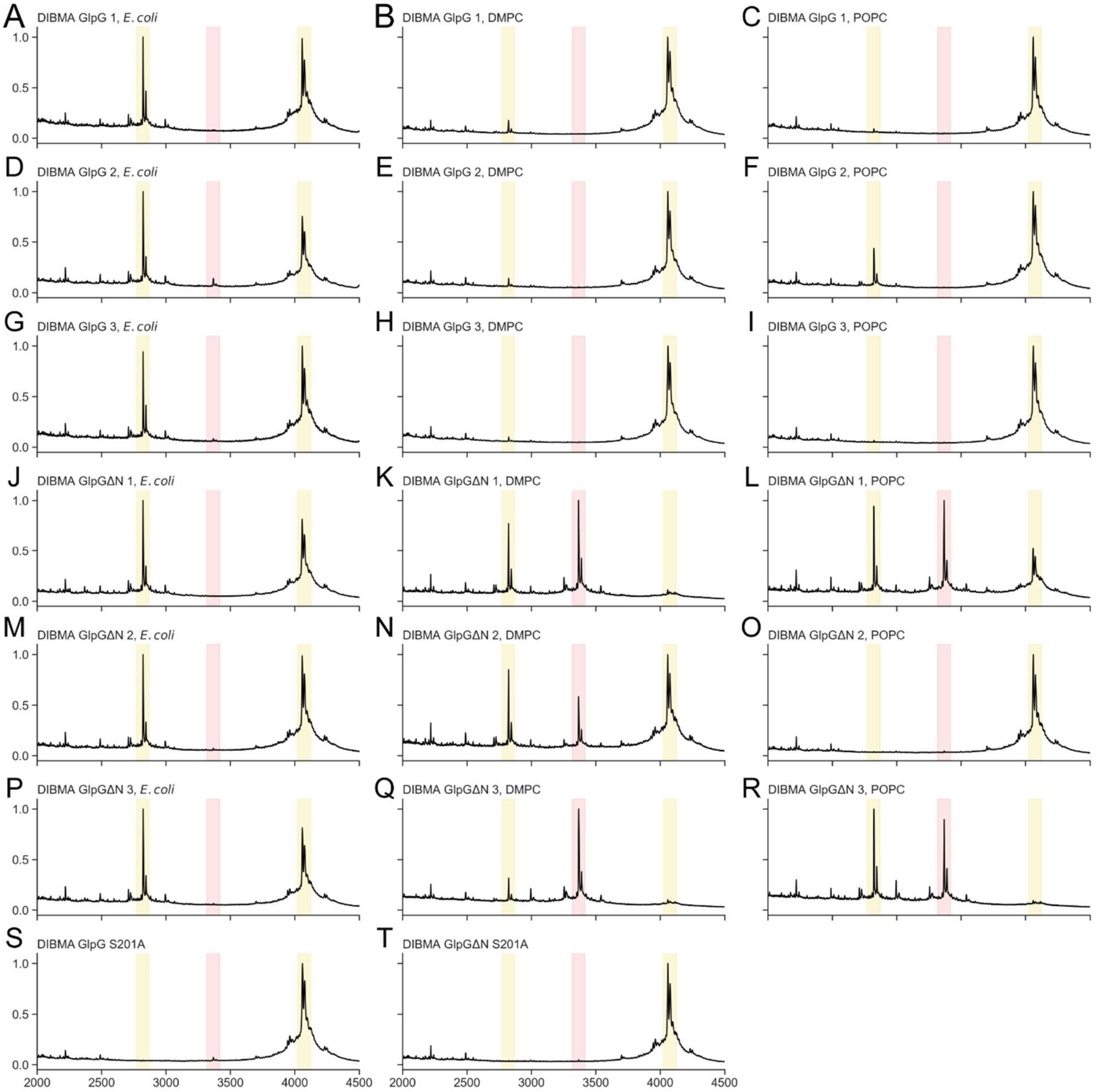

Supplementary Figure 6 – Normalized MALDI-TOF spectra after 24 hours incubation of TatA:GlpG in a 5:1 molar ratio from DIBMA-solubilized GlpG and GlpGΔN. (A-I, S) MALDI spectra of TatA-FITC cleavage by GlpG and associated inactive S201A mutant solubilized by DIBMA into natively ('Initial', left column) extracted membrane DIBMALPs, post DMPC wash ('DMPC', centre column), or post POPC wash ('POPC', right column). (J-R,T) MALDI spectra of TatA-FITC cleavage by GlpGΔN and associated inactive S201A mutant solubilized by DIBMA into natively ('Initial', left column) extracted membrane DIBMALPs, post DMPC wash ('DMPC', centre column), or post POPC wash ('POPC', right column). Numbering indicates biological repeats. Yellow highlighted regions denote expected regions of either cleaved TatA peptide (left, ~2800 Da), or un-cleaved TatA-FITC (right, ~4100 Da). Red highlighted region denotes unexpected cleavage product, with a calculated mass of 3368 Da. S and T show MALDI spectra for TatA incubated with inactive mutant S201A of GlpG and GlpGΔN, respectively.

Supplementary Table 1 – Mean relative head group composition (as percentage of total lipid) of full-length (wild type) GlpG, expressed in C41 BL21 (DE3) *E. coli* cells, and GlpGΔN, expressed in C43 BL21 (DE3) *E. coli* cells. Errors shown are standard deviation (DM solubilized sample n=2, DIBMA solubilized sample n=3). For uninduced/induced cells and S201A samples no biological repeats were performed. Lipid head groups are shortened where PC = phosphatidylcholine, PE = phosphatidylethanolamine, PG = phosphatidylglycerol, PS = phosphatidylserine, CL = cardiolipin.

	GlpG (C41)				GlpGΔN (C43)			
**Sample**	**%PC**	**%PE**	**%PG**	**%CL**	**%PC**	**%PE**	**%PG**	**%CL**
Uninduced cells	0.0	76.2	12.4	11.3	0.1	81.4	13.3	5.3
Induced cells	0.0	59.8	18.3	21.8	0.2	77.6	13.6	8.7
GlpG (DM)	0.8 (±0.1)	56.2 (±5.2)	17.2 (±1.2)	25.8 (±3.8)	0.4 (±0.0)	64.8 (±7.6)	13.1 (±1.5)	21.6 (±9.1)
GlpG (DIBMA)	0.8 (±0.5)	55.3 (±4.2)	21.3 (±0.8)	22.6 (±4.5)	0.1 (±0.1)	69.0 (±0.7)	10.3 (±2.9)	20.5 (±3.7)
GlpG (DIBMA) +DMPC	61.3 (±10.1)	22.1 (±5.8)	8.9 (±2.2)	7.7 (±2.2)	45.7 (±9.3)	37.6 (±9.0)	5.4 (±0.5)	11.4 (±4.0)
GlpG (DIBMA) +POPC	66.4 (±10.6)	18.6 (±5.4)	7.0 (±2.3)	8.1 (±3.1)	22.3 (±5.2)	50.2 (±5.4)	7.6 (±0.9)	19.9 (±3.7)
GlpG S201A (DM)	0.4	47.1	14.8	37.7	1.2	62.6	9.9	26.2
GlpG S201A (DIBMA)	0.3	46.3	19.5	33.9	0.5	62.6	13.2	23.7
GlpG S201A (DIBMA) +DMPC	71.5	14.7	6.5	7.2	45.8	32.6	5.7	16.0
GlpG S201A (DIBMA) +POPC	56.2	21.1	9.7	12.9	19.3	46.5	9.2	25.0

Supplementary Table 2 – Relative composition (as percentage total lipid) for most commonly retained lipids after DMPC/POPC washing. Lipids shown by initial head group and carbon chain identifiers. Memb. sample denotes whole-cell extract from induced cells. Errors shown are standard deviation (DM solubilized sample n=2, DIBMA solubilized sample n=3). For Memb. samples no biological repeats were performed.

	GlpG (C41)					GlpGΔN (C43)				
**Sample**	**Memb.**	**DM**	**DIBMA**	**DMPC**	**POPC**	**Memb.**	**DM**	**DIBMA**	**DMPC**	**POPC**
**PE 30:0**	2.5	2.3 (±0.4)	2.5 (±0.3)	0.9 (±0.2)	0.7 (±0.2)	5.5	5.2 (±0.3)	5.4 (±1.0)	2.7 (±0.6)	3.7 (±0.1)
**PE 32:1**	12.2	10.0 (±1.8)	8.8 (±1.1)	3.5 (±1.2)	2.4 (±0.6)	11.3	7.2 (±0.2)	7.3 (±0.6)	3.7 (±1.3)	5.0 (±0.9)
**PE 33:1**	12.5	11.3 (±1.5)	12.8 (±1.0)	5.0 (±1.4)	3.5 (±1.4)	29.7	22.5 (±2.9)	23.9 (±0.8)	12.2 (±4.0)	16.6 (±2.7)
**PE 34:1**	13.6	9.8 (±1.4)	10.0 (±0.5)	4.1 (±1.2)	3.0 (±0.9)	8.3	6.5 (±2.0)	8.7 (±0.9)	4.7 (±1.6)	6.2 (±1.5)
**PE 34:2**	4.2	4.8 (±0.6)	3.9 (±0.2)	1.7 (±0.4)	1.2 (±0.5)	2.9	3.2 (±0.3)	3.5 (±0.3)	1.8 (±0.4)	2.5 (±0.1)
**PE 35:2**	3.5	3.6 (±0.0)	4.2 (±0.4)	1.6 (±0.5)	1.3 (±0.4)	5.6	5.9 (±1.0)	6.3 (±0.1)	3.3 (±0.7)	4.6 (±0.4)
**PE 36:2**	4.7	4.2 (±0.1)	4.7 (±0.5)	2.0 (±0.6)	1.5 (±0.5)	2.5	3.2 (±0.2)	4.0 (±0.1)	2.1 (±0.7)	2.8 (±0.4)
**PG 34:1**	7.1	5.8 (±0.2)	7.5 (±0.5)	3.2 (±0.8)	2.5 (±0.8)	3.1	3.0 (±0.5)	2.9 (±0.6)	1.4 (±0.2)	2.2 (±0.1)
**PG 36:2**	3.5	3.8 (±0.4)	4.6 (±0.1)	2.0 (±0.5)	1.7 (±0.5)	1.5	1.9 (±0.4)	1.6 (±0.4)	0.9 (±0.1)	1.1 (±0.2)
**CL 66:2 (32:1/34:1)**	4.0	2.3 (±1.0)	2.4 (±0.3)	0.9 (±0.3)	0.8 (±0.1)	1.8	2.9 (±1.9)	3.3 (±0.6)	1.3 (±0.4)	3.1 (±0.9)
**CL 70:3 (34:1/36:2)**	4.0	4.4 (±0.4)	4.6 (±1.4)	1.4 (±0.3)	1.4 (±0.7)	1.1	2.2 (±0.8)	3.4 (±0.7)	1.8 (±0.8)	3.4 ±(0.8)

Supplementary Table 3 – Total lipid and protein amounts (in mol) used in lipidomic analysis, per sample. Numbers indicate biological repeats, and are congruent with similar sample numbering in this work. The averaged values come from the initial purification values for both GlpG and GlpGΔN.

Sample Name	Lipid (pmol)	Lipid (mol)	Protein (μM)	Protein used (mol)	Ratio (lipid/protein)
**GlpG (DM) 1**	99.99	1.00E-10	12.72	1.27E-11	7.9
**GlpG (DM) 2**	136.17	1.36E-10	15.83	1.58E-11	8.6
**GlpGΔN (DM) 1**	161.29	1.61E-10	19.1	1.91E-11	8.4
**GlpGΔN (DM) 2**	183.96	1.84E-10	34.9	3.49E-11	5.3
**GlpG S201A (DM)**	278.85	2.79E-10	6.57	6.57E-12	42.4
**GlpGΔN S201A (DM)**	102.11	1.02E-10	9.24	9.24E-12	11.1
**GlpG S201A DIBMA**	480.22	4.80E-10	14.9	1.49E-11	32.2
**GlpGΔN S201A DIBMA purified**	291.92	2.92E-10	13.75	1.38E-11	21.2
**GlpG DIBMA initial purification 1**	579.42	5.79E-10	16.8	1.68E-11	34.5
**GlpG DIBMA initial purification 2**	270.10	2.70E-10	9.4	9.40E-12	28.7
**GlpG DIBMA initial purification 3**	285.28	2.85E-10	11.2	1.12E-11	25.5
**GlpG DIBMA 1 DMPC wash**	780.90	7.81E-10	15.23	1.52E-11	51.3
**GlpG DIBMA 1 POPC wash**	184.43	1.84E-10	3.1	3.10E-12	59.5
**GlpG DIBMA 2 DMPC wash**	122.02	1.22E-10	3.61	3.61E-12	33.8
**GlpG DIBMA 2 POPC wash**	998.40	9.98E-10	14.55	1.46E-11	68.6
**GlpG DIBMA 3 DMPC wash**	428.00	4.28E-10	6.21	6.21E-12	68.9
**GlpG DIBMA 3 POPC wash**	274.04	2.74E-10	6.03	6.03E-12	45.4
**GlpGΔN DIBMA initial purification 1**	1453.34	1.45E-09	30.05	3.01E-11	48.4
**GlpGΔN DIBMA initial purification 2**	3475.99	3.48E-09	84.91	8.49E-11	40.9
**GlpGΔN DIBMA initial purification 3**	409.69	4.10E-10	20.22	2.02E-11	20.3
**GlpGΔN DIBMA 1 DMPC wash**	309.89	3.10E-10	10.05	1.01E-11	30.8
**GlpGΔN DIBMA 1 POPC wash**	341.54	3.42E-10	12.31	1.23E-11	27.7
**GlpGΔN DIBMA 2 DMPC wash**	407.05	4.07E-10	11.5	1.15E-11	35.4
**GlpGΔN DIBMA 2 POPC wash**	1196.66	1.20E-09	27.64	2.76E-11	43.3
**GlpGΔN DIBMA 3 DMPC wash**	117.52	1.18E-10	4.94	4.94E-12	23.8
**GlpGΔN DIBMA 3 POPC wash**	115.31	1.15E-10	6.43	6.43E-12	17.9
**GlpG S201A DMPC**	841.03	8.41E-10	10.87	1.09E-11	77.4
**GlpG S201A POPC**	695.89	6.96E-10	13.17	1.32E-11	52.8
**GlpGΔN S201A DMPC**	34.78	3.48E-11	3.41	3.41E-12	10.2
**GlpGΔN S201A POPC**	74.56	7.46E-11	10.26	1.03E-11	7.3
**Average DM initial**	145.35	1.45E-10	20.6375	2.06E-11	7.0
**Average DIBMA initial**	1078.97	1.08E-09	28.76	2.88E-11	37.5
