# Supplementary material for: Lipid-polymer nanoparticles to probe the native-like environment of intramembrane rhomboid protease GlpG and its activity
Source: Nat Commun. 2024 Aug 30;15:7533. doi: 10.1038/s41467-024-51989-0 (PMC11364529; doi:10.1038/s41467-024-51989-0)
Supplement: Supplementary file 4 — Source Data [file 41467_2024_51989_MOESM4_ESM.zip › Source data/Gel images (uncropped).pdf]

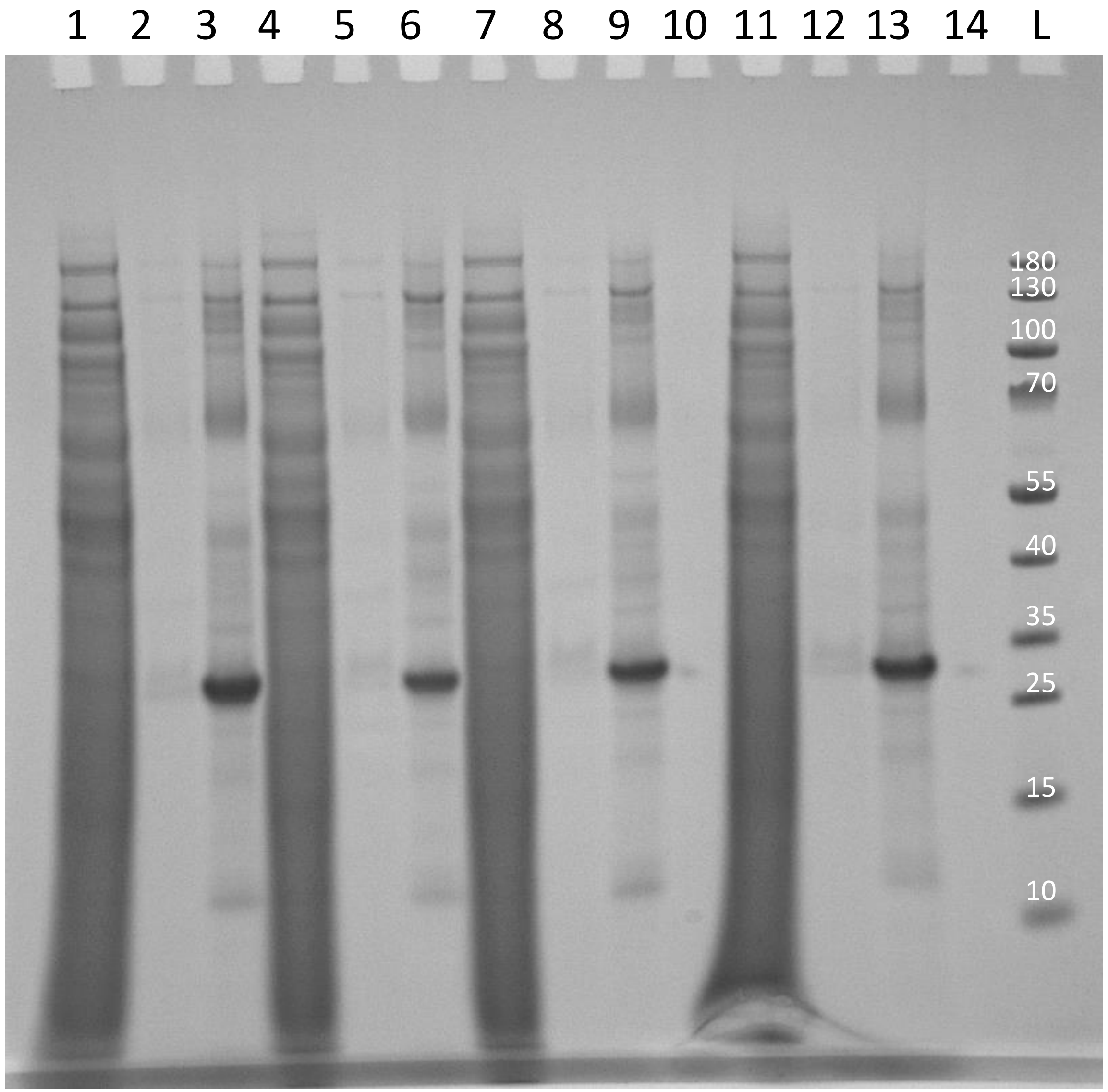

GlpG FL DIBMA initial Ni-NTA

1. FL 1 F.T
2. FL 1 W
3. FL 1 Elution
4. FL 2 F.T
5. FL 2 W
6. FL 2 Elution
7. FL 3 F.T
8. FL 3 W
9. FL 3 Elution
10. -
11. S201A FL F.T
12. S201A FL W
13. S201A FL Elution

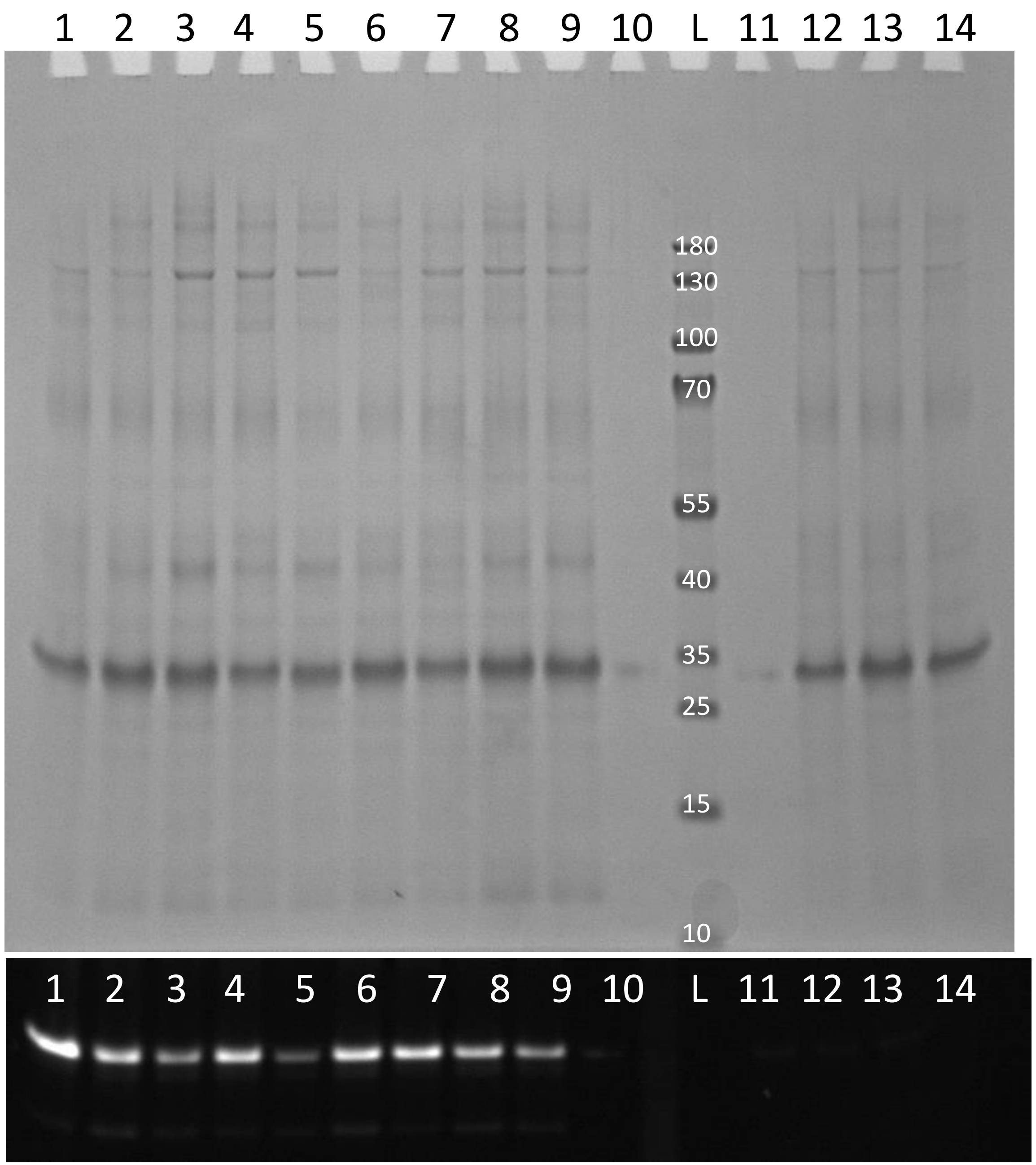

GlpG FL DIBMA TAMRA-FP labelling

1. FL 1 initial
2. FL 1 DMPC
3. FL 1 POPC
4. FL 2 initial
5. FL 2 DMPC
6. FL 2 POPC
7. FL 3 initial
8. FL 3 DMPC
9. FL 3 POPC
10. -
11. Ladder
12. -
13. S201A initial
14. S201A DMPC
15. S201A POPC

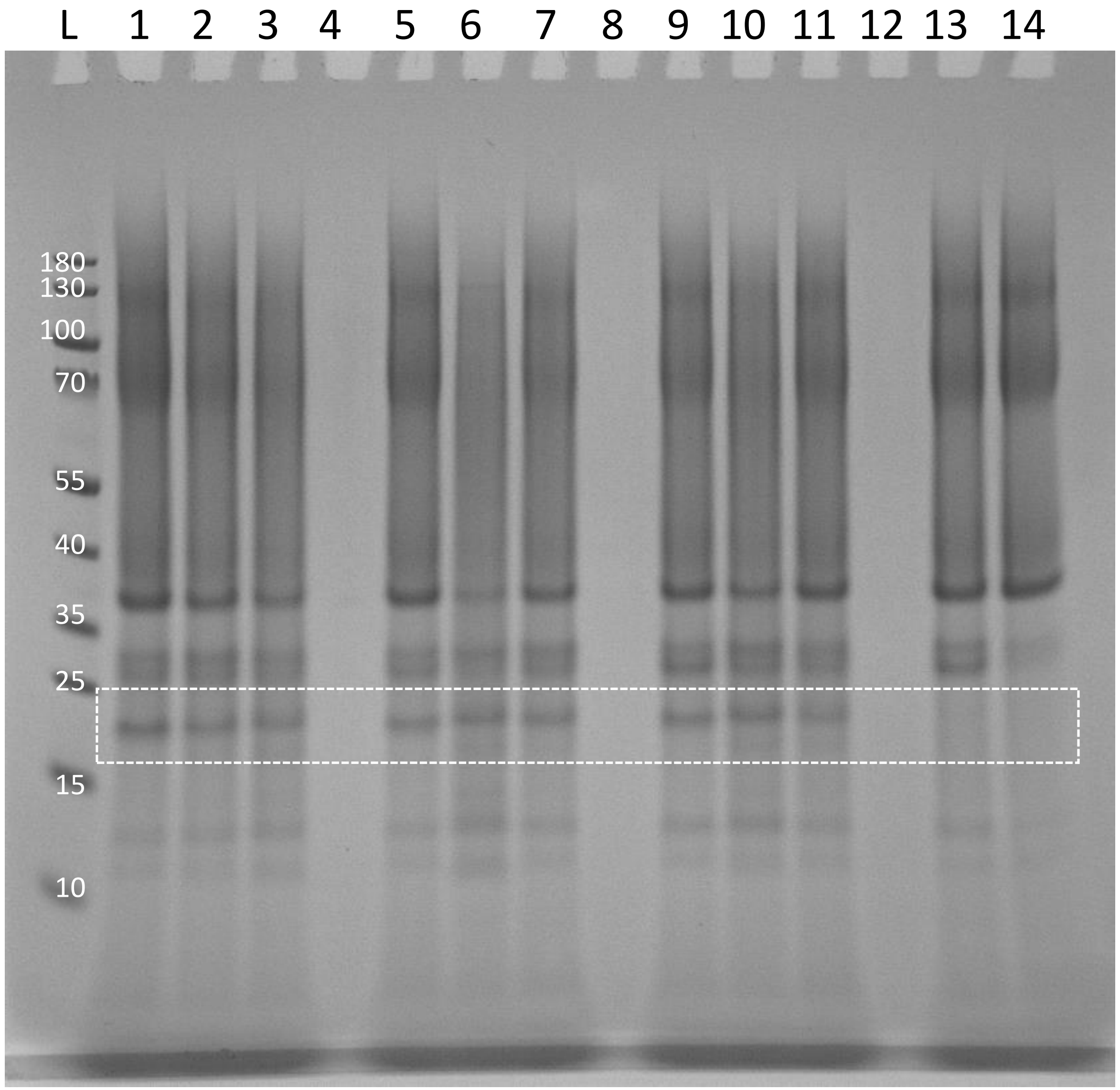

GlpG FL DIBMA SUMO-TatA-FLAG cleavage

1. FL 1 initial
2. FL 1 DMPC
3. FL 1 POPC
4. -
5. FL 2 initial
6. FL 2 DMPC
7. FL 2 POPC
8. -
9. FL 3 initial
10. FL 3 DMPC
11. FL 3 POPC
12. -
13. S201A initial
14. SUMO-TatA-FLAG control

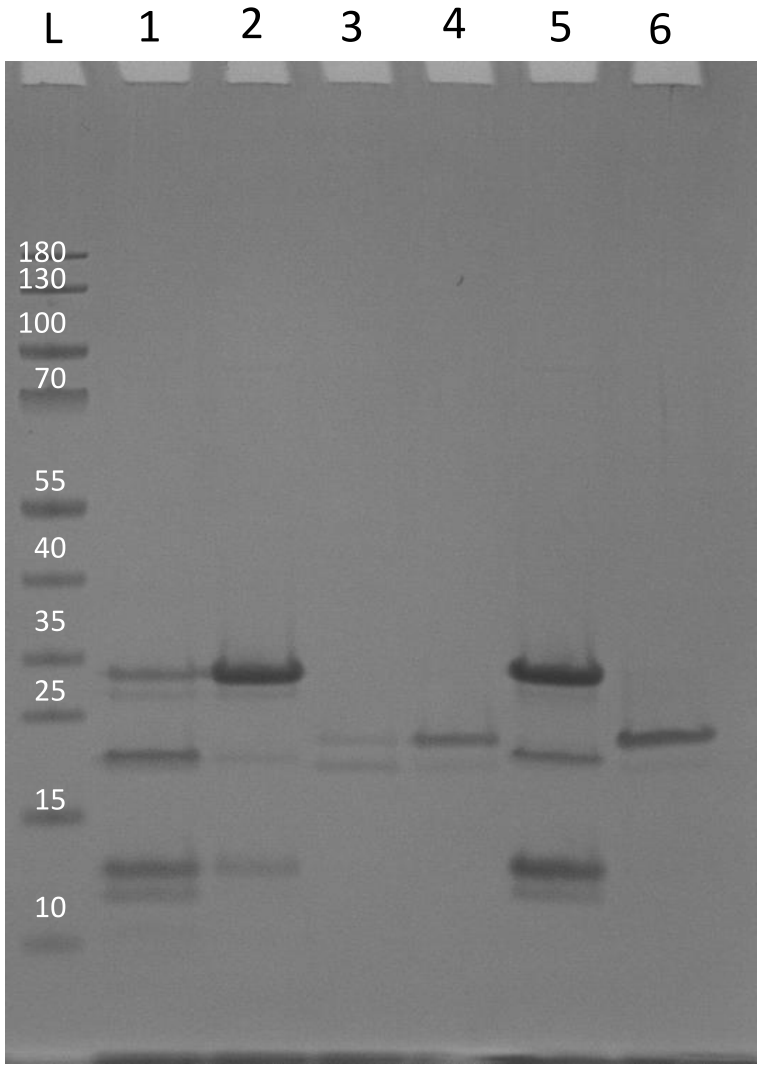

GlpG DM purity gel

1. FL 1
2. FL 2
3. dN 1
4. dN 2
5. S201A F.L
6. S201A dN

GlpG dN DIBMA TAMRA-FP labelling

1. dN 1 initial
2. dN 1 DMPC
3. dN 1 POPC
4. dN 2 initial
5. dN 2 DMPC
6. dN 2 POPC
7. dN 3 initial
8. dN 3 DMPC
9. dN 3 POPC
10. Ladder
11. dN S201A initial
12. dN S201A DMPC
13. dN S201A POPC

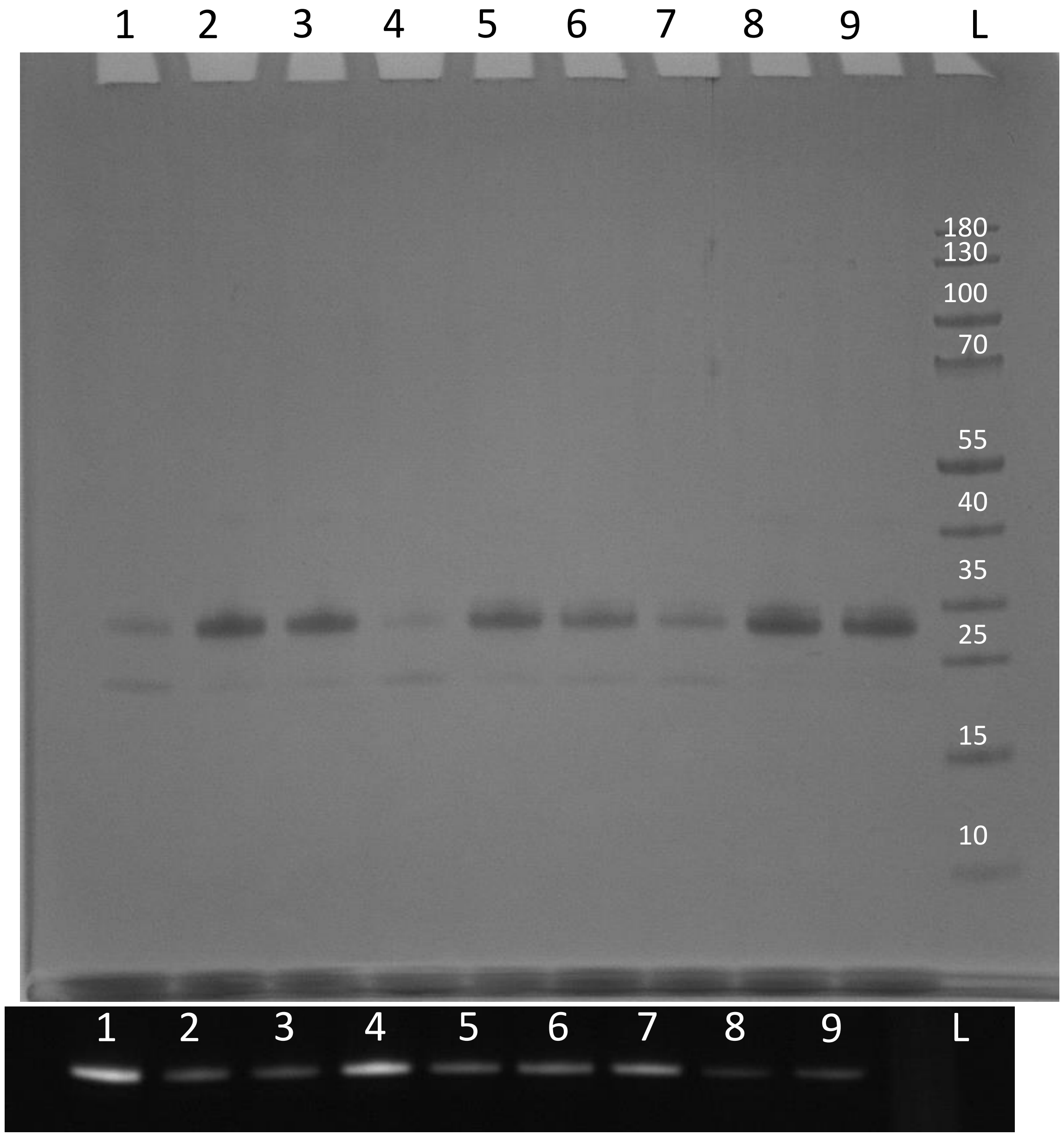

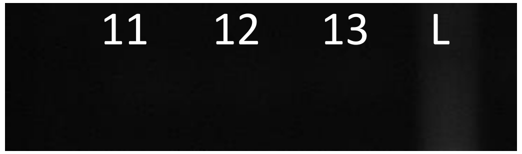

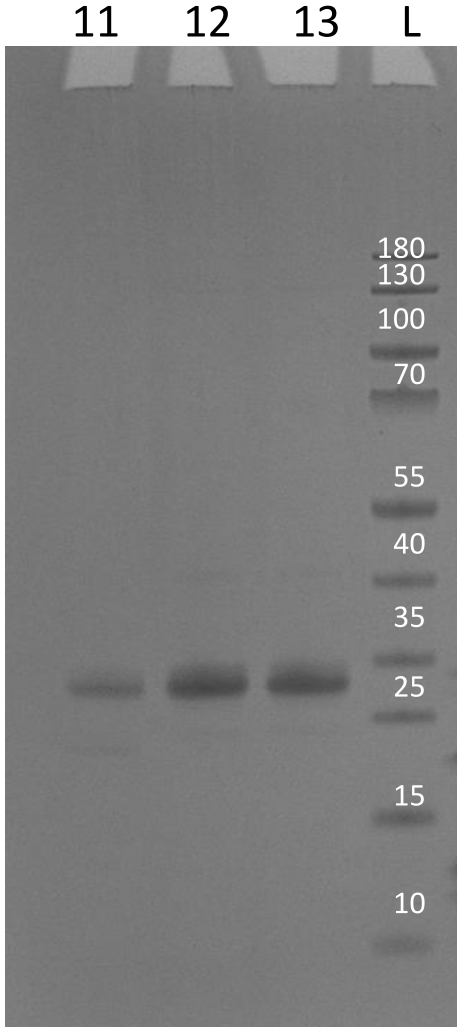

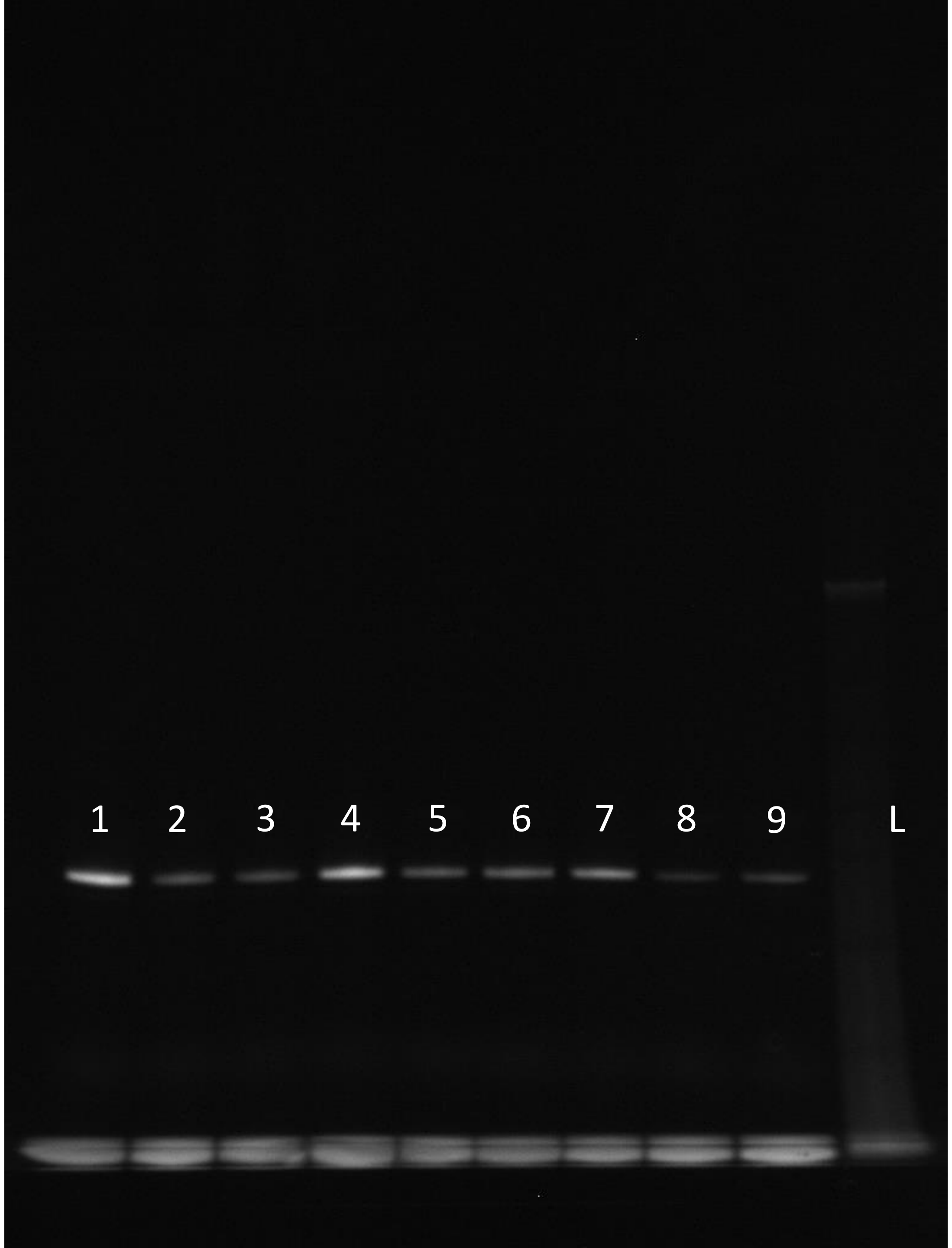

GlpG dN DIBMA TAMRA-FP labelling

1. FL 1 initial
2. FL 1 DMPC
3. FL 1 POPC
4. FL 2 initial
5. FL 2 DMPC
6. FL 2 POPC
7. FL 3 initial
8. FL 3 DMPC
9. FL 3 POPC
10. Ladder

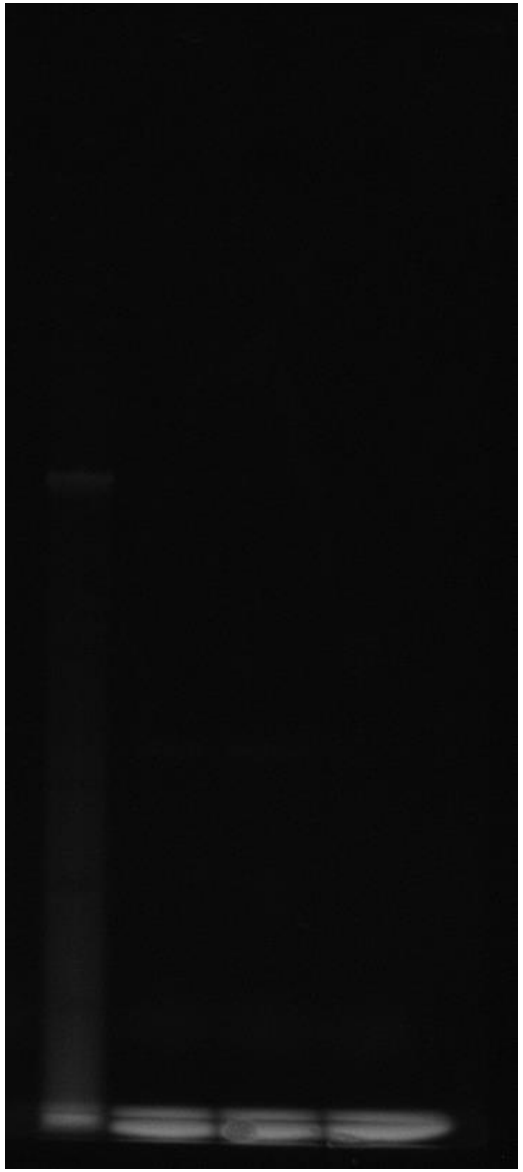

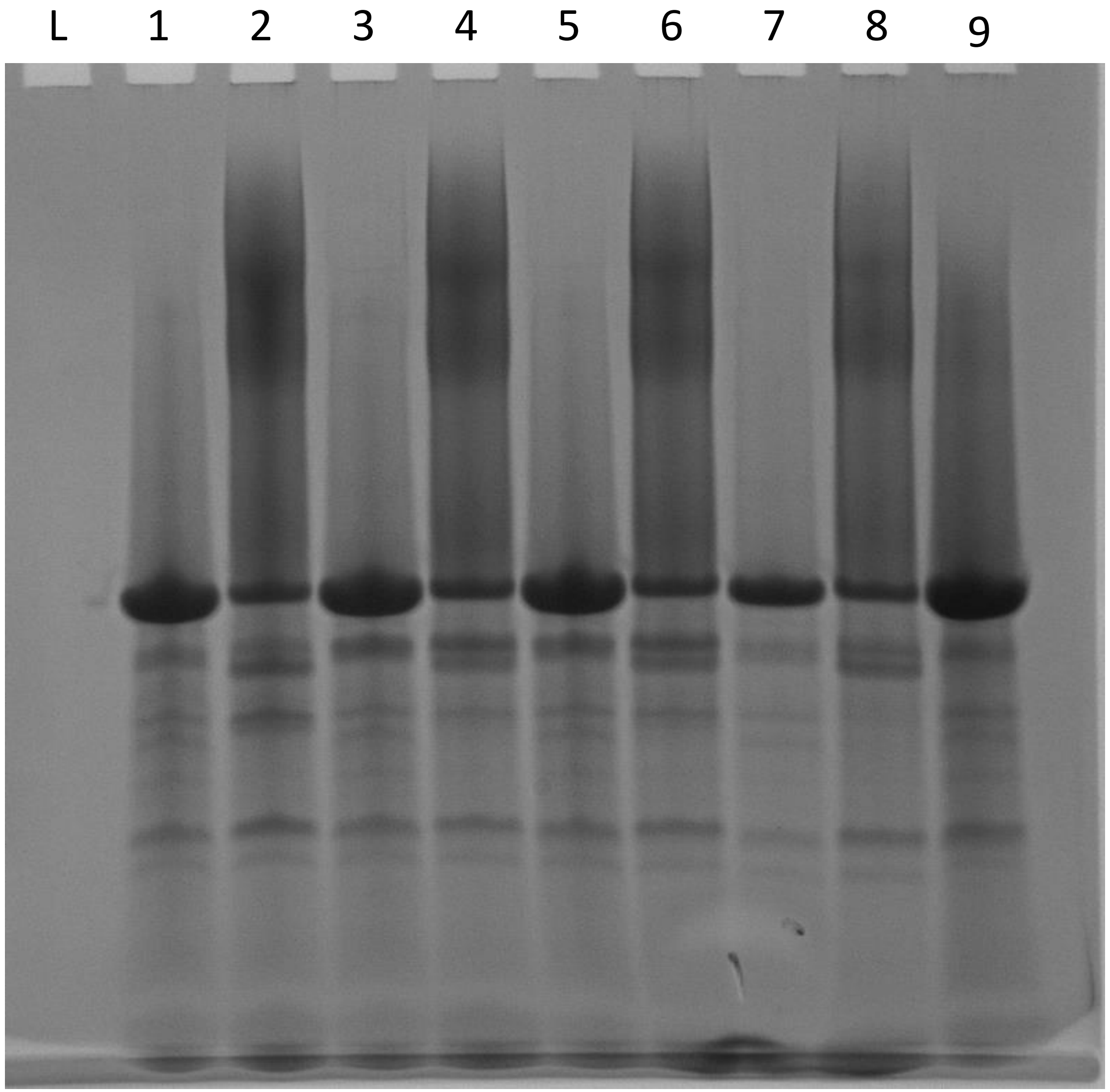

GlpG dN DIBMA SUMO-TatA-FLAG cleavage

1. dN 1 initial
2. dN 1
3. dN 1 DMPC initial
4. dN 1 DMPC
5. dN POPC initial
6. dN POPC
7. dN S201A initial
8. dN S201A
9. TatA control

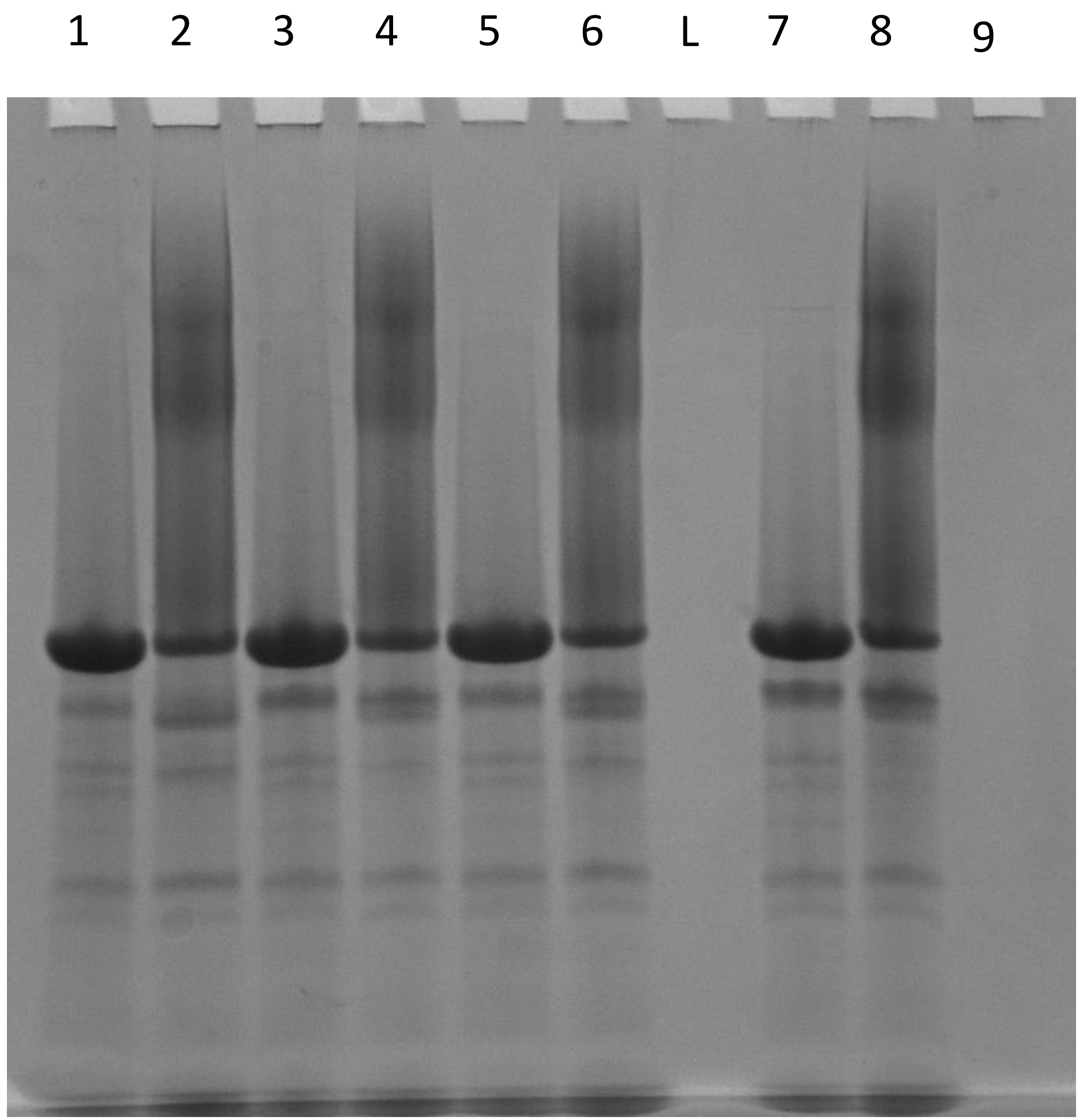

GlpG dN DIBMA SUMO-TatA-FLAG cleavage

1. dN 2 initial
2. dN 2
3. dN 2 DMPC initial
4. dN 2 DMPC
5. dN 2 POPC initial
6. dN 2 POPC
7. -
8. dN S201A DMPC initial
9. dN S201A DMPC

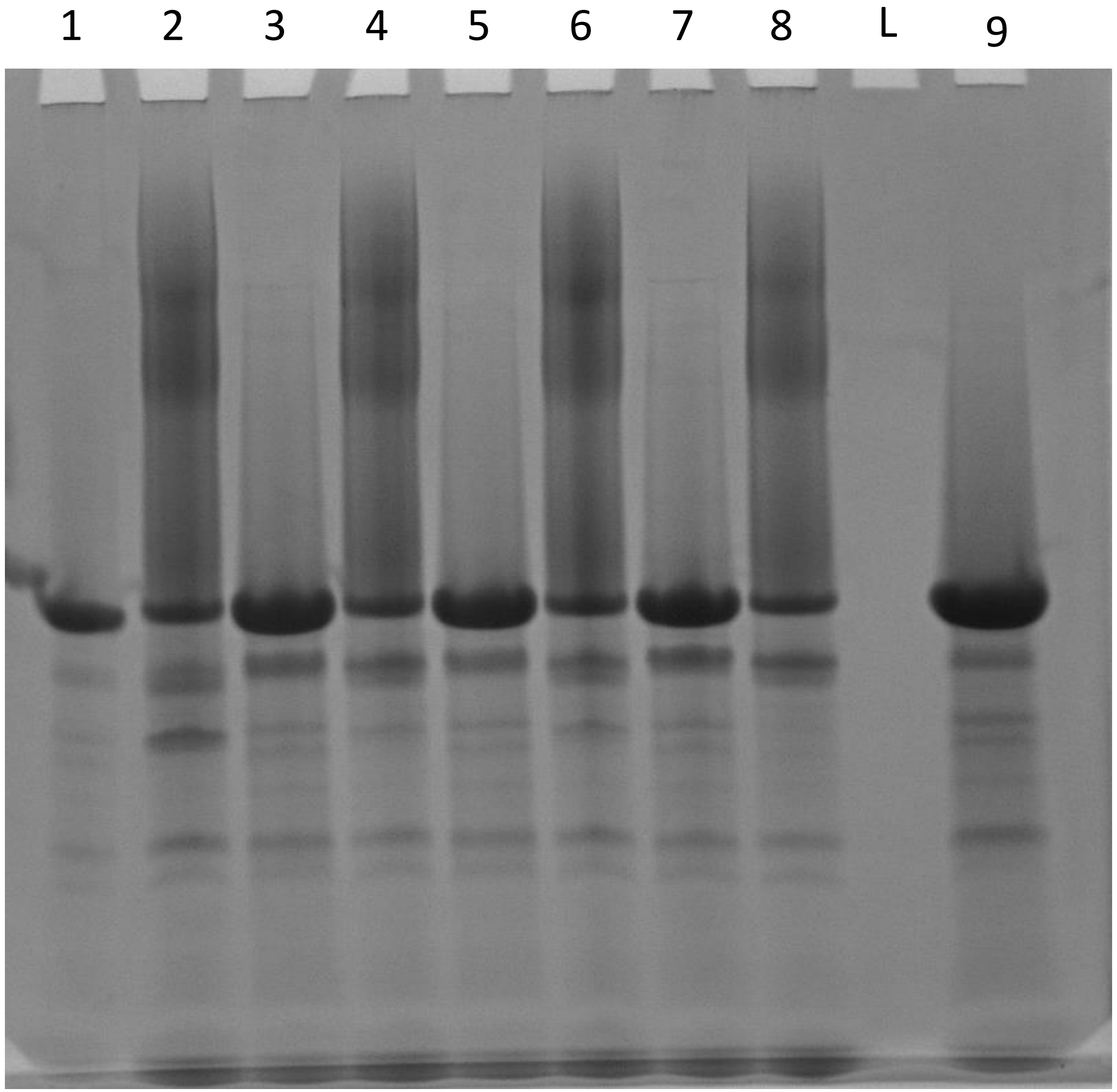

GlpG dN DIBMA SUMO-TatA-FLAG cleavage

1. dN 3 initial
2. dN 3
3. dN 3 DMPC initial
4. dN 3 DMPC
5. dN 3 POPC initial
6. dN 3 POPC
7. dN S201A POPC initial
8. dN S201A POPC
9. -
10. TatA cont. initial

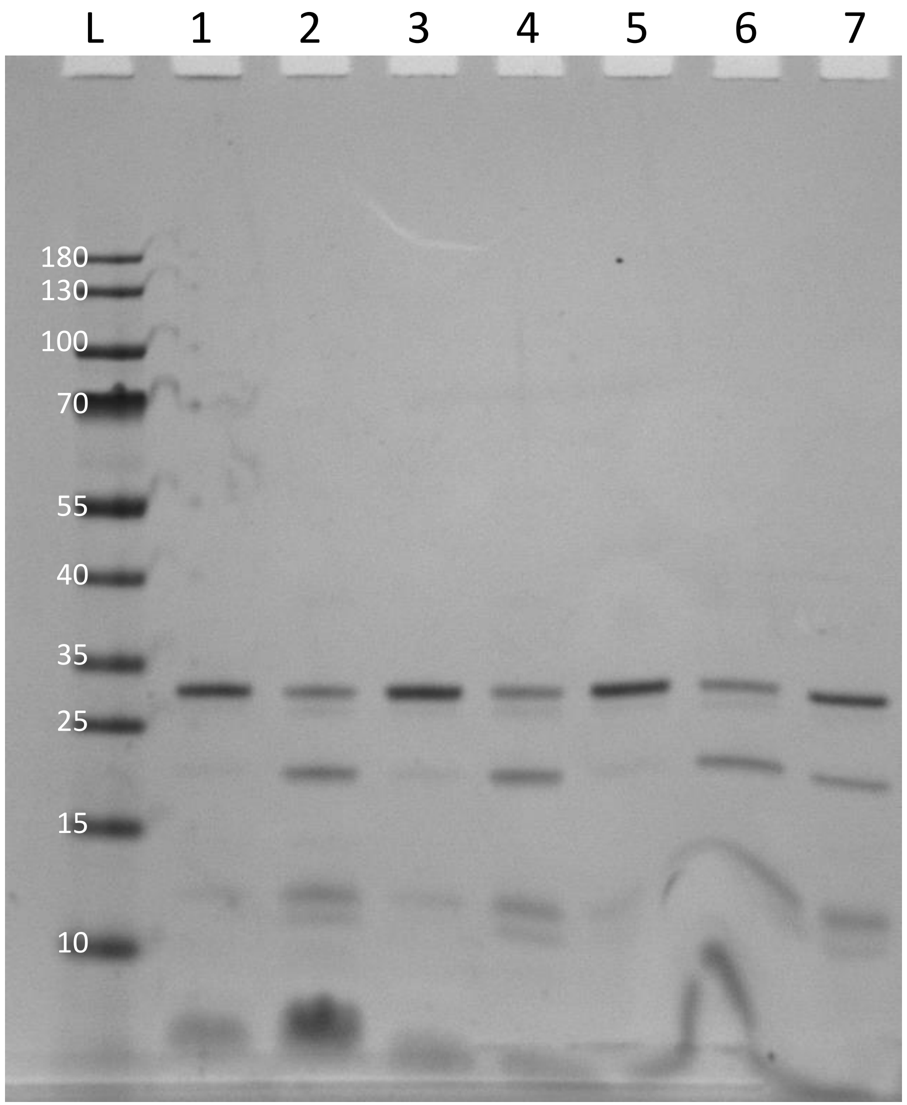

GlpG FL (DM solubilized) TAMRA labelling

1. GlpG FL 1, *E. coli*
2. GlpG FL 2, *E. coli*
3. GlpG FL 1, DMPC
4. GlpG FL 2, DMPC
5. GlpG FL 1, POPC
6. GlpG FL 2, POPC
7. GlpG FL S201A, *E. coli*

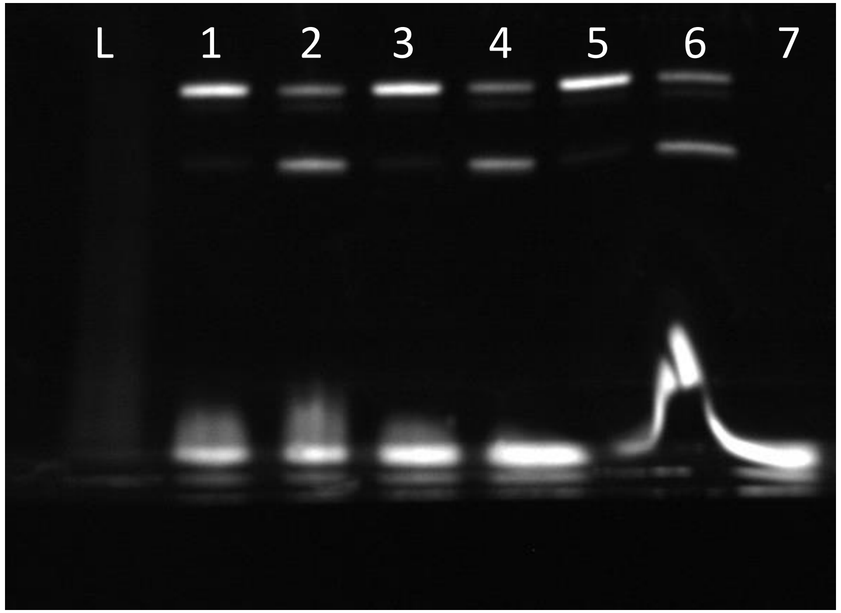

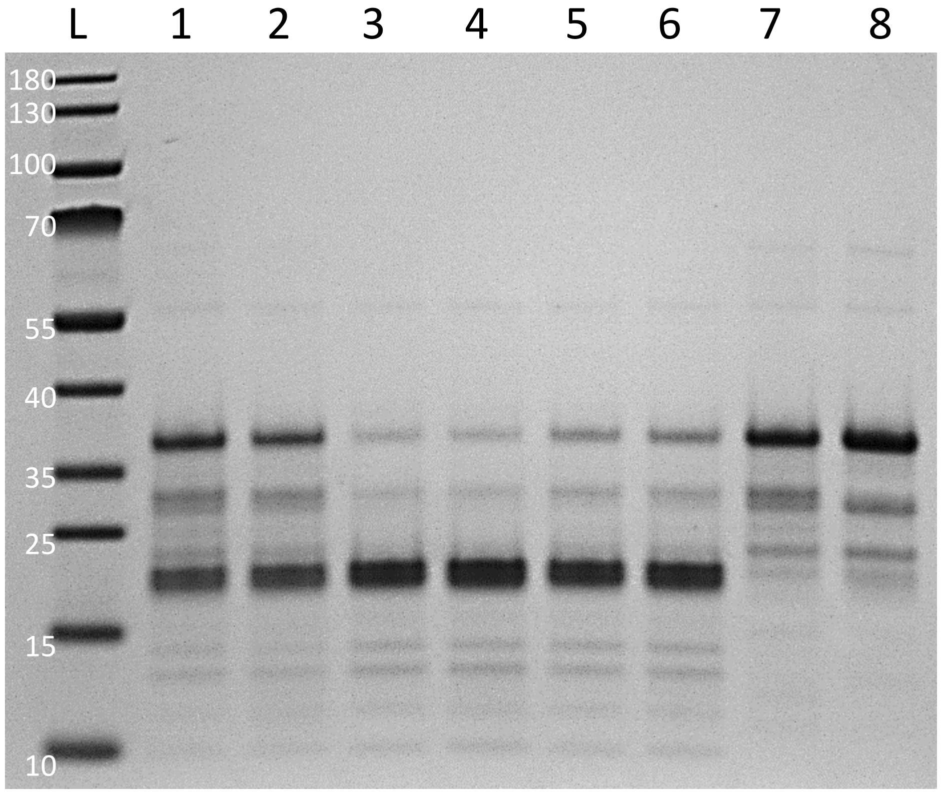

GlpG FL (DM solubilized) SUMO-TatA cleavage

1. GlpG FL 1, *E. coli*
2. GlpG FL 2, *E. coli*
3. GlpG FL 1, DMPC
4. GlpG FL 2, DMPC
5. GlpG FL 1, POPC
6. GlpG FL 2, POPC
7. GlpG FL S201A, *E. coli*
8. *SUMO-TatA only*

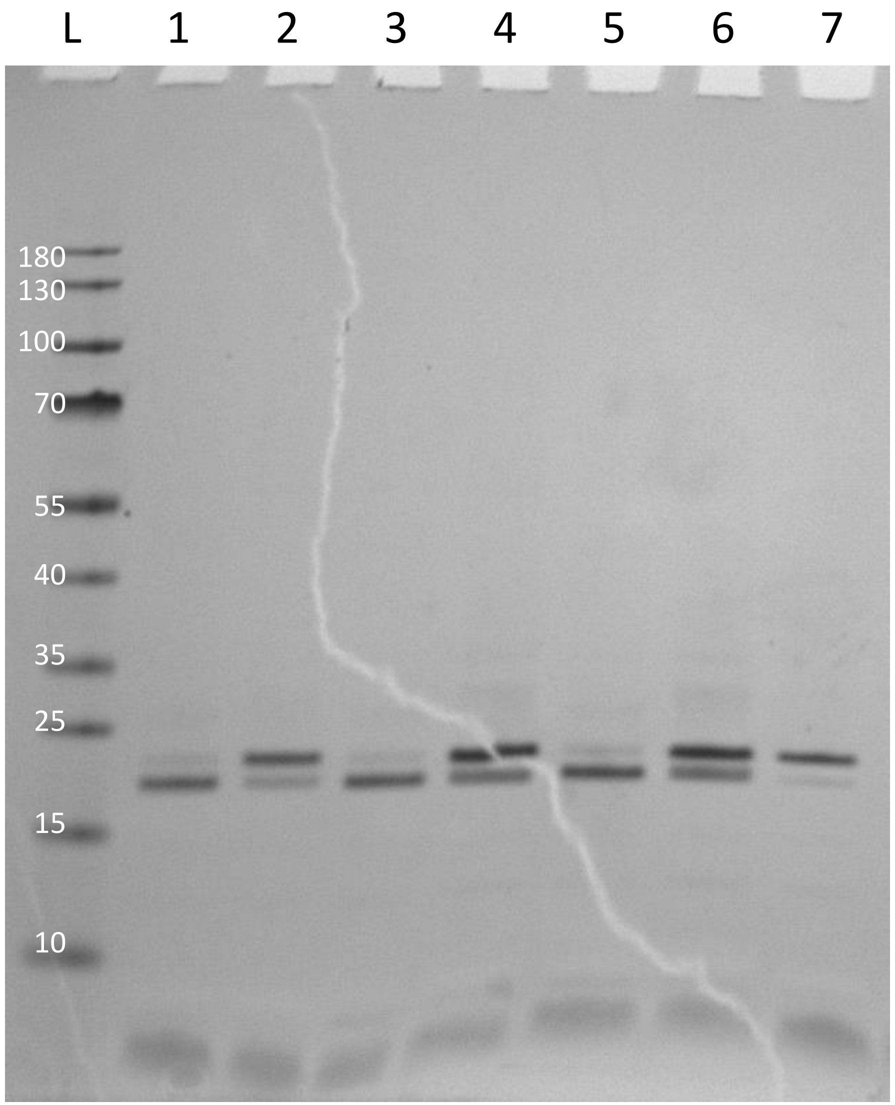

GlpG dN (DM solubilized) TAMRA labelling

1. GlpG dN 1, *E. coli*
2. GlpG dN 2, *E. coli*
3. GlpG dN 1, DMPC
4. GlpG dN 2, DMPC
5. GlpG dN 1, POPC
6. GlpG dN 2, POPC
7. GlpG dN S201A, *E. coli*

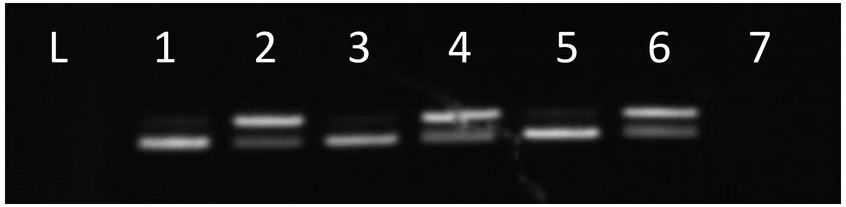

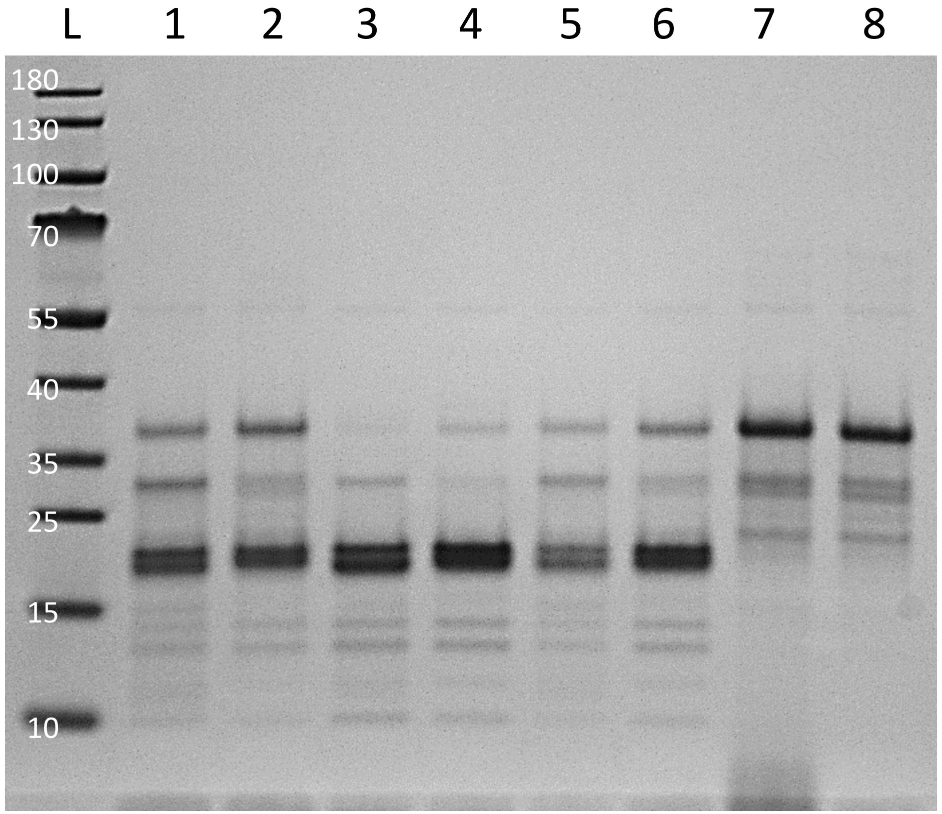

GlpG dN (DM solubilized) SUMO-TatA

1. GlpG dN 1, *E. coli*
2. GlpG dN 2, *E. coli*
3. GlpG dN 1, DMPC
4. GlpG dN 2, DMPC
5. GlpG dN 1, POPC
6. GlpG dN 2, POPC
7. GlpG dN S201A, *E. coli*
8. *TatA-SUMO only*
